# 

**Published:** 1883-01

**Authors:** 


### BOOK NOTICES.

THE MEDICAL RECORD VISITING LIST, OR PHYSICIANS DIARY FOR 1883. New York: Wm. Wood & Co.

This elegant pocket record appears fully furnished forth with things indispensable to the busy doctor, in compact brevity.

BLAKISTON'S VISITING LIST.

The oldest book of the kind well-known to the profession, needs no comment. It contains about the same memoranda as usual, with the addition of the dosage of the rarest and newest drugs.

THE LAS VEGAS MEDICAL SOCIETY, of New Mexico, held their annual meeting at the Plaza Hotel,
Vice President Dr. M. W. Robbins in the chair.

On motion, reports of the secretary and treasurer were read and accepted.

On motion, the society decided that no vote by proxy would be accepted.

The society then proceeded to the election of officers for the ensuing year. The presiding officer appointed Doctors Peebles and Milligan tellers. The following officers were unanimously elected: Dr. W. H. Page, president; Dr. M. W. Robbins, vice president; Dr. W. H. Ashley, secretary; Dr. R. Baily, treasurer; Dr. M. M. Milligan, librarian.

THE EIGHTH SESSION of the International Medical Congress will take place in Copenhagen from the 10th to the 16th of August, 1884.

### CLINICS.

MONDAY.

Eye and Ear Infirmary—1.15 to 2.15 p. m., Otological, by Dr. Schaefer; 2.15 to 3.30 p. m., Ophthalmological, Dr. Holmes.
Mercy Hospital—2 p. m., Medical, Profs. Hollister and Quine.
Rush Medical College—3 p. m., Dermatological and Venereal, by Prof. Hyde.
Woman's Medical College—2 p. m., Dermatological and Venereal, by Prof. Maynard.

TUESDAY.

Cook Co. Hospital—2 to 4 p. m., Medical and Surgical Clinics.
Mercy Hospital—2 p. m., Surgical Clinic, by Prof. Andrews.
Woman's Medical College—10 a. m., Prof. Ingals.

WEDNESDAY.

Chicago Medical College—2 p. m., Eye and Ear, by Prof. Jones.
Rush Medical College—2 p. m., Medical by Prof. Bridge; 3 p. m., Ophthalmological and Otological, by Prof. Holmes: 3 to 4 p. m., Diseases of the Chest, by Prof. Ross.
Woman's Medical College—2 p. m., Eye and Ear, by Dr. W. T. Montgomery; 3 p. m., Diseases of Children, by Prof. Chas. W. Earle.
Eye and Ear Infirmary—2.30 p. m.. Dr. E. J. Gardiner.

THURSDAY.

Chicago Medical College—2 p. m., Gynæcological, Prof. Dudley.
Rush Medical College—2 p. m., Diseases of Children, by Prof. Knox; 3 p. m., Diseases of the Nervous System, by Prof. Lyman.
Eye and Ear Infirmary—1.15 to 2.25 p. m., Otological, by Dr. Schaefer; 2.15 to 3.30, p. m., Ophthalmological, Dr. Holmes.
Woman's Medical College—3 p. m., Surgical, by Prof. Owens.
College of Physicians and Surgeons—2 p. m., Medical, by Prof. S. A. McWilliams; 3 p. m., Surgical, by Prof. R. L. Rea.

FRIDAY.

Cook County Hospital—2 to 4 p. m., Medical and Surgical Clinics.
Mercy Hospital—2 p. m., Medical, by Prof. Davis.

SATURDAY.

Rush Medical College—2 p. m., Surgical, by Prof. Gunn.
Mercy Hospital—2 p. m., Surgical Clinic, by Prof. Andrews.
Chicago Medical College—3 p. m., Neurological, Prof. Jewell.
Woman's Medical College—2 p. m., Gynæcological, by Prof. Stevenson.
College of Physicians and Surgeons—2 p. m., Diseases of the Chest, by Prof. S. A. McWilliams; 3 p. m., Gynæcological, by Prof. A. Reeves Jackson.

Daily Clinics, from 2 to 4 p. m., at the Central Free Dispensary, at the South Side Dispensary and at the West Side Dispensary.

